# Multi-Sensor Respiratory–Swallow Telecare System for Safe Feeding in Different Trunk Inclinations: System Development and Clinical Application

**DOI:** 10.3390/s23020642

**Published:** 2023-01-06

**Authors:** Wann-Yun Shieh, Chin-Man Wang, Yan-Ying Ju, Hsin-Yi Kathy Cheng

**Affiliations:** 1 Department of Computer Science and Information Engineering, College of Engineering, Chang Gung University, No. 259, Wen-Hwa 1st Road, Kwei-Shan, Taoyuan 333, Taiwan; 2 Department of Physical Medicine and Rehabilitation, Chang Gung Memorial Hospital, 5 Fu-Hsing Street, Kwei-Shan, Taoyuan 333, Taiwan; 3 Collage of Medicine, Chang Gung University, Taoyuan 333, Taiwan; 4 Department of Adapted Physical Education, National Taiwan Sport University, No. 250, Wen-Hwa 1st Road, Kwei-Shan, Taoyuan 333, Taiwan; 5 Graduate Institute of Early Intervention, College of Medicine, Chang Gung University, No. 259, Wen-Hwa 1st Road, Kwei-Shan, Taoyuan 333, Taiwan

**Keywords:** feeding, positioning, swallowing, breathing, biomedical, sensors

## Abstract

Proper positioning is especially important to ensure feeding and eating safely. With many nursing facilities restricting visitations and close contact during the coronavirus pandemic, there is an urgent need for remote respiratory–swallow monitoring. This study aimed to develop a semiautomatic feeding telecare system that provides instant feedback and warnings on-site and remotely. It also aimed to analyze the effects of trunk positions on respiratory–swallow coordination. A signal collector with multiple integrated sensors for real-time respiratory–swallow monitoring and warning was developed. A repeated measures design was implemented to evaluate the effects of trunk inclination angles on the swallow-related functions. Significant differences in inclination angles were discovered for swallowing apnea (*p* = 0.045) and total excursion time of thyroid cartilage (*p* = 0.037), and pairwise comparisons indicated that these differences were mostly present at 5° to 45°. Alerts were triggered successfully when undesired respiratory patterns or piecemeal occurred. The results indicated that a care recipient can swallow more easily when sitting upright (5°) than when leaning backward (45°). This telecare system provides on-site and remote respiratory–swallow monitoring and alerting for residents in care facilities and can serve as a pipeline for the early screening of swallowing dysfunction.

## 1. Introduction

Long-term care is increasingly required as populations begin to age. Between 10% and 20% of individuals who are older than 65 years are estimated to have swallowing difficulties [1,2]. Furthermore, individuals with multiple disabilities also have problems controlling their muscle tone and maintaining their posture. In particular, these individuals experience problems during feeding or eating. Therefore, feeding or assisted eating is a key daily routine for nursing institutions. A study revealed that call lights in nursing homes were used most frequently before and after mealtimes, indicating that staff assistance is most required during this period [3]. In the context of nursing care, the smoothness of feeding and effective swallowing directly affect the nutrient intake and quality of life of these individuals [4].

Swallowing and breathing cannot occur concurrently. When swallowing starts, breathing stops. This ensures that during the act of eating, food does not stray into the trachea and cause inhalation choking. Breathing does not resume until food passes through the pharynx and reaches the esophagus [5,6,7]. A typical symptom of swallowing dysfunction is residual food or liquid on the swallowing path, which tends to cause choking, aspiration, or potential complications. If this condition is treated inadequately, swallowing dysfunction and its complications can cause dehydration, malnutrition, aspiration pneumonia, or even death [8,9]. Problems relating to muscle tone and posture maintenance also increase the difficulty of maintaining a sitting or reclining posture when eating. This problem can even interfere with feeding/eating and swallowing movements. Only coordinated swallowing and breathing functions can ensure a smooth feeding process.

An appropriate seat-back and bed-board inclination angle benefits the feeding or eating process [10]. Positioning is regarded as a simple, effective, and noninvasive method that can be applied to a care recipient during feeding [11]. A reclining position facilitates the gravity-driven flow of bolus or liquid from the mouth to the pharynx [11,12]. The ideal inclination angle for smooth and effective swallowing during feeding is difficult to determine [13]. The optimal inclination angles can change depending on disease condition, food form or texture, and the physical environment. Although care recipients usually communicate their discomfort, institutionalized older adults or individuals with disabilities may not have the linguistic ability required to express themselves clearly. Consequently, if a caregiver is inattentive during feeding or under time constraints and fails to detect their care recipient’s reaction, the choking risks caused by feeding are considerably increased. Especially in the coronavirus (COVID-19) pandemic, most institutions restrict visitations and close contact between residents and caregivers [14,15,16]. Therefore, remote monitoring in lieu of in-person monitoring is more important. A respiratory–swallow monitoring and warning mechanism should be implemented to ensure the safety of care.

To evaluate respiratory–swallow coordination, videofluoroscopic swallow study (VFSS) has been most frequently used [6,17]. X-ray was used in VFSS for monitoring the swallowing events, especially those related to the hyoid bone and thyroid cartilage movements. Another method is fiberoptic endoscopic evaluation of swallowing (FEES) [18]. During FEES, a fiberoptic endoscope is inserted through the nasal cavity to the pharynx to obtain images of the swallowing process. However, both VFSS and FEES are invasive, and a VFSS also introduces radiation risks. In addition, performing a VFSS or FEES for patients at risk of dysphagia is impractical because a healthcare facility may not have the required equipment [19]. Triaxial accelerometers have also been used to measure pharynx movement, but a misjudgment may occur when the acceleration generated by the swallowing movement of a patient with dysphagia is overly small [20]. Considering the advantages and disadvantages of existing measurement methods, our team used surface electromyography (sEMG) for submentalis and piezoelectric sensors (force sensing resistor, FSR) to measure the movement of the thyroid cartilage and measured nasal airflow by using a nasal cannula [21,22]. Through the time locking of the signals from the three sensors on a frame, respiratory–swallow coordination can be evaluated accurately and efficiently in a noninvasive manner. An autodetection program with a customized algorithm was developed to conduct signal processing and capture temporal parameters. This program was verified to be viable and reliable for capturing and analyzing temporal parameters during a swallowing movement [8]. The previous analytic respiratory–swallow model is used only for offline analyses; it is still incapable of providing instant feedback or warnings to a caregiver.

The aim of the study was to develop a portable, noninvasive, multi-sensor semiautomatic feeding monitoring system with a respiratory–swallow analyzer and instant warning indicators, and to analyze the effects of various trunk inclination positions and various food types on respiratory–swallow coordination.

## 2. Materials and Methods

### 2.1. System Development

**Figure 1:**
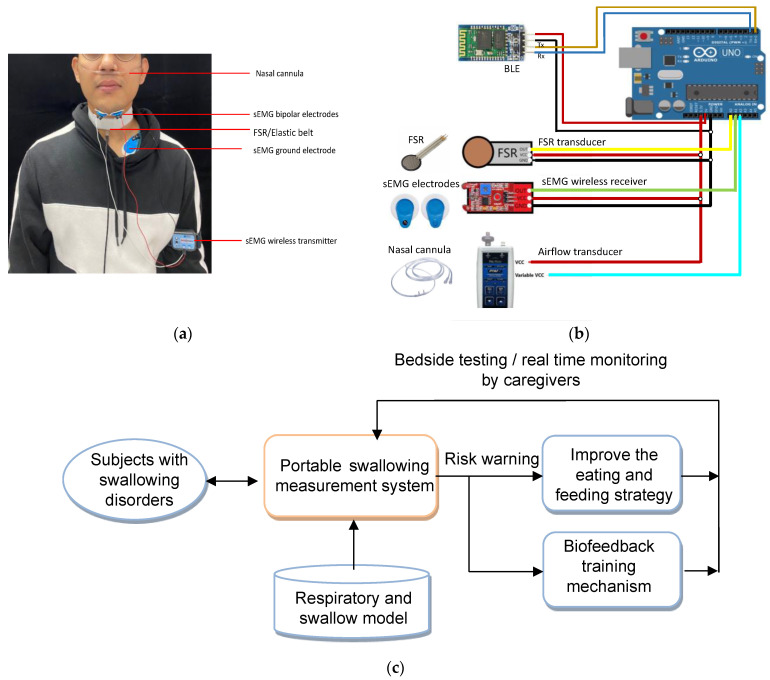
(**a**) Positions of each sensor: sEMG, nasal airflow, and FSR; (**b**) the circuit of the designed signal collector; (**c**) the real-time respiratory–swallowing telecare model.

An integrated multi-sensor signal collector with real-time respiratory–swallow monitoring and warning functions was developed (Figure 1). Figure 1a illustrates the attached position and usage of each sensor, including sEMG, nasal airflow, and FSR. The aforementioned sensors were integrated into a portable signal collector to develop remote monitoring applications. Figure 1b illustrates the circuit of the designed signal collector, and Figure 1c illustrates the real-time respiratory-swallowing telecare model.

### 2.2. Multisensor-Based Analytical Swallowing Model

**Figure 2:**
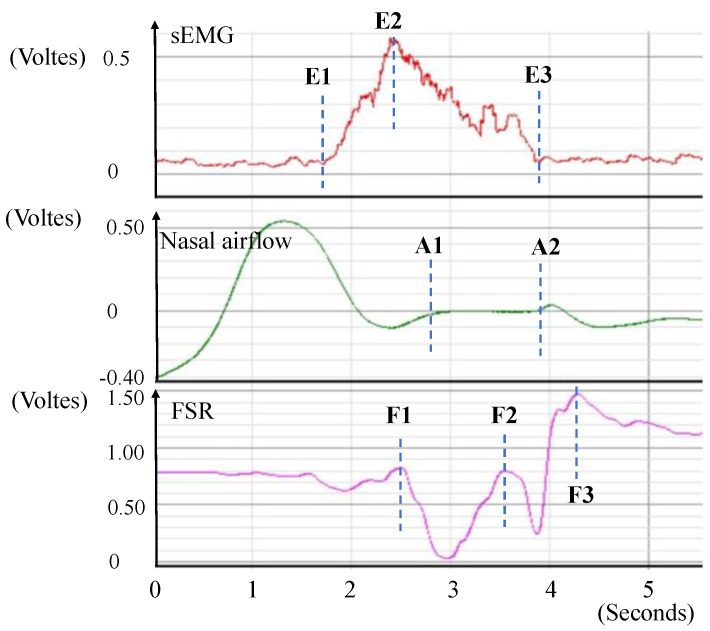
Swallowing and respiration signals from three sensors.

Figure 2 presents the synchronized signals that were detected by the sensors when a normal participant swallowed 10 mL of room-temperature water. The sEMG waveform indicated submentalis activity during swallowing (E1 to E3), with the largest force being recorded at E2. Synchronously, the nasal airflow waveform indicated a sign of apnea (A1 to A2). This was regarded as a protective phenomenon that allows for safe swallowing without aspiration. The FSR waveform typically displays a W-shaped response to represent the two phases of thyroid cartilage excursion. The first phase (F1 to F2) represents the movement of the thyroid cartilage upward and forward to block the trachea and ensure that water smoothly passes through the pharynx to the esophagus. The second phase (F2 to F3) represents the movement of the thyroid cartilage back to its original position. Table 1 lists the variables that describe a complete swallowing process, and they can be used to measure the latency, duration, and amplitude among the related signals. Two possible respiratory phases were observed before and after the respiration apnea period, namely, expiration (EXP) and inspiration (INP). These entail four possible respiratory patterns: EXP/EXP, EXP/INP, INP/EXP, and INP/INP. Only EXP/EXP was regarded as a physiologically safe swallowing pattern.

The occurrence of the feature points in Figure 2 (i.e., A1–A2, E1–E3, and F1–F3) can be detected automatically by monitoring the signal patterns along the time sequence. In our previous study [9], a slope-based method has been developed that involves monitoring the slope variation of the signal curve in a sliding window to verify whether the waveform fits the predefined swallowing patterns. This slope-based method has been implemented in the proposed portable signal collector (Figure 1b). Nine variables were monitored in this study (Table 1). The current method can run in real-time with minimal delay. The variable patterns registered with this portable system were validated by comparing those from the BIOPAC MP 150 (BIOPAC systems Inc., Goleta, CA, USA), a medical instrument widely used for data acquisition and display. The average response delay of the portable system was controlled within 20 ms.

### 2.3. Warning Mechanism

Swallowing disorders can be detected by monitoring the respiratory and swallowing variables. Table 2 lists two risk levels for swallowing disorders that relate to safe feeding and to which caregivers should be alerted. Level 1 is primarily triggered by two events, namely, piecemeal deglutition and long apnea duration (L_SAD). When the volume of water or bolus exceeds a specific limit, a participant may have insufficient strength to swallow the bolus in one gulp and divide it into smaller pieces for swallowing (i.e., piecemeal deglutition). In this situation, a caregiver should pause the feeding process. Otherwise, the bolus that remains in the oral or pharyngeal position may block the respiratory path and cause choking. The same situation may occur when the apnea duration (i.e., SAD in Table 1) of swallowing is longer than a specific period. Feeding can continue when the aforementioned events cease, when the individual returns to a stable condition.

The Level 2 alert is triggered by the abnormal presentation of other variables. Successful swallowing requires smooth coordination among nasal, oral, and pharyngeal positions. Thus, a large gap between each onset time point (i.e., E1 and F1 in Table 1), a long duration of physiological response in swallowing (i.e., D_sEMG_, TET, and D_2DEF_), or unsafe respiratory phases before and after respiration apnea (i.e., non-EXP/EXP Resp_phase) may indicate a swallowing disorder. In these situations, a caregiver should reduce the volume of the bolus or the speed of feeding.

**Figure 3:**
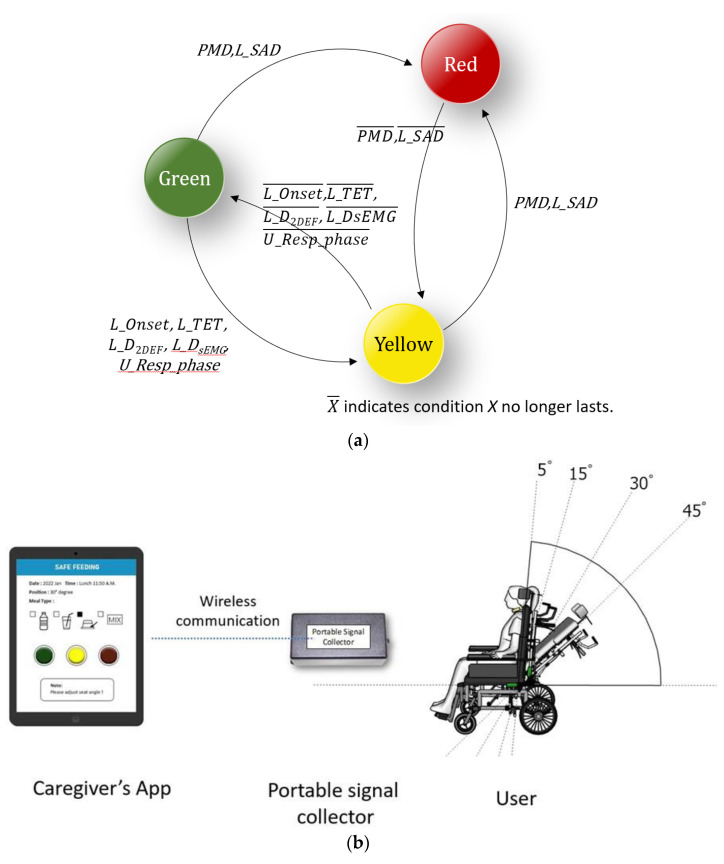
(**a**) A state diagram that illustrates the warning mechanism; (**b**) an example of the remote respiratory–swallowing monitoring when a participant is sitting and adopting various trunk inclination positions while swallowing.

Based on the risk levels, a warning mechanism was proposed. Level 1 events present a greater level of swallowing risk than Level 2 events. Thus, alerts of Level 1 and Level 2 events are color coded as red and yellow, respectively. When the situations that previously triggered an alert are no longer detected, the alert light changes to green, indicating that a participant has returned to their initial or normal state. Figure 3a is a state diagram that illustrates this warning mechanism. This warning mechanism can be implemented through an app that was designed to be used by caregivers in a practical setting. Figure 3b provides an example of the use of this application when a participant is sitting and adopting various trunk inclination positions while swallowing. A caregiver can receive the alert and monitor the respiratory–swallowing states remotely through telecommunication.

### 2.4. Clinical Evaluation

The aforementioned portable collection and analysis system was used to evaluate the effects of trunk inclination angles on respiratory–swallow monitoring. Thirty-three participants who were aged between 21 and 50 years and did not display any signs of swallowing difficulty were included. All participants provided informed consents in accordance with the regulations of Chang Gung Memorial Hospital (IRB number 201600752A3). The clinical trial registration number was NCT05221801. The aforementioned sensors were applied to the participants in accordance with the procedures described in an earlier section of the present study. The proposed portable signal collector and a caregiver’s app were used for data acquisition.

A reclining wheelchair (Guardian, Karma) was used to enable adjustments to inclination angle. The backrest of this wheelchair provided the required degree of back recline (5° to 45°). The two arms of an electrogoniometer were attached to the sides of the seat and the backrest and connected to the caregiver’s app to register the inclination angles. Four angles (5°, 15°, 30°, and 45°) were evaluated. When a participant leaned back, their head could rest on the headrest of the wheelchair. The neck of the participant was kept in a neutral (0° extension) position when they leaned back on the headrest. The examiner then located the anatomic markers for swallowing and respiration signal recording. Before sensors were attached to a participant, their submental muscles had to be in a relaxed state. Three food types (specifically, 1 mL water, 10 mL water, and 5 mL pudding) were used to examine the swallowing movements of the participant. A combination of seat inclination (four levels) and food type (three levels) was randomly assigned, with three repetitions being performed for each combination.

Before a test was conducted, water (measuring 5 mL) was first given to a participant in the upright position to allow them to familiarize themselves with the test procedures. At this time, the examiner also verified whether the swallowing movement of the participant triggered the green light on the signal collector. During the data collection process, water was measured and drawn using identical, disposable 20 mL syringes and given to participants in identical, small disposable cups. The formal test was then conducted after a 3 min rest. Each participant performed 36 swallows and rested for at least 30 s between each swallow. If the participant displayed any sign of tracheal aspiration (e.g., choking, coughing more than twice, or shortness of breath) during the test, the procedures were halted. During each swallowing test, the participants held the water/pudding in their mouths until a verbal cue was given for them to initiate the desired swallow. The variable measurements are listed in Table 1.

Statistical analyses were performed using SPSS 22 software (SPSS, Chicago, IL, USA). Descriptive statistics were used to describe the basic features of the data. Repeated measure analyses of variance were used to analyze the differences among the dependent variables concerning seat inclination and food content combination. Bonferroni pairwise multiple comparisons were performed when significant differences were detected. An alpha value of 0.05 was regarded as statistically significant.

## 3. Results

**Figure 4:**
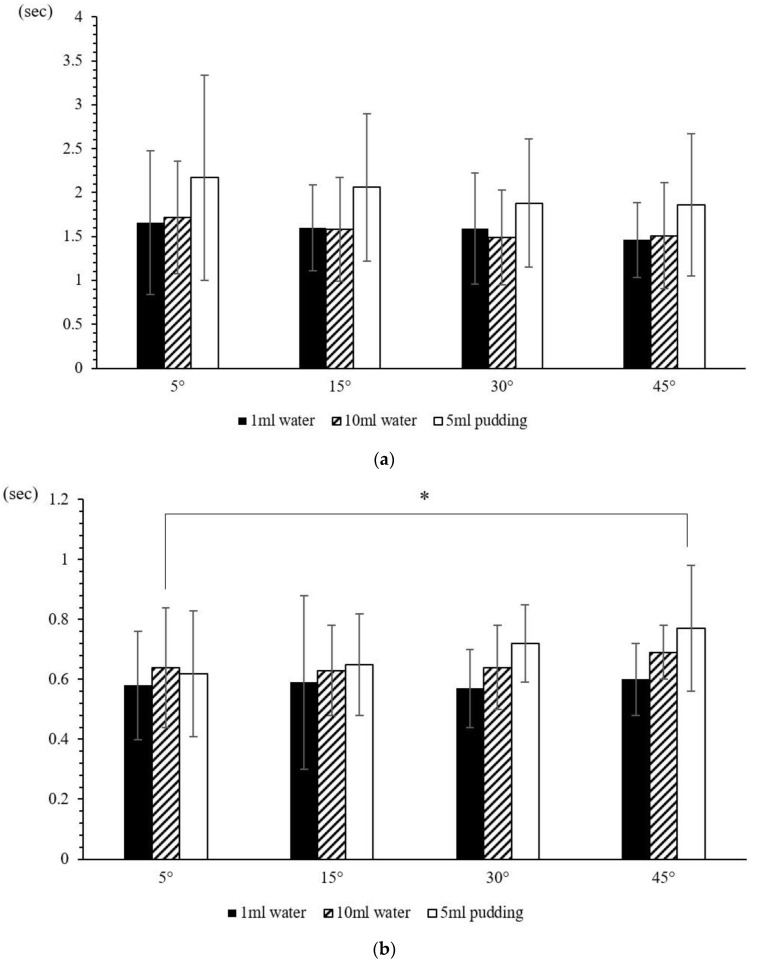

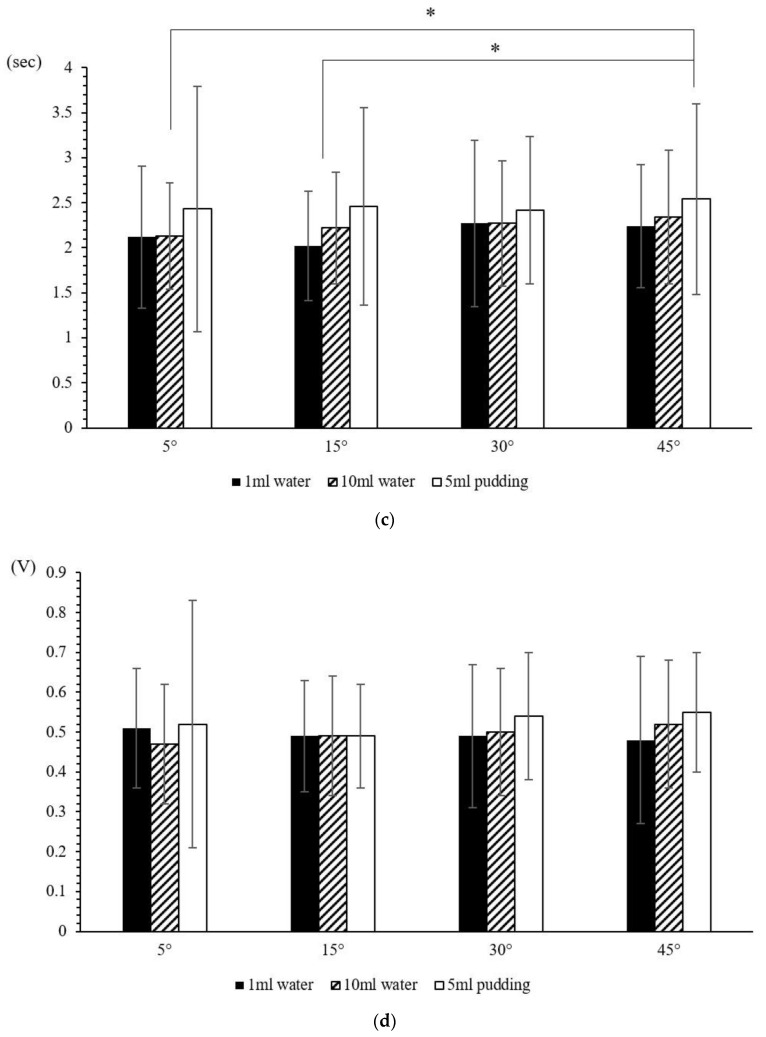

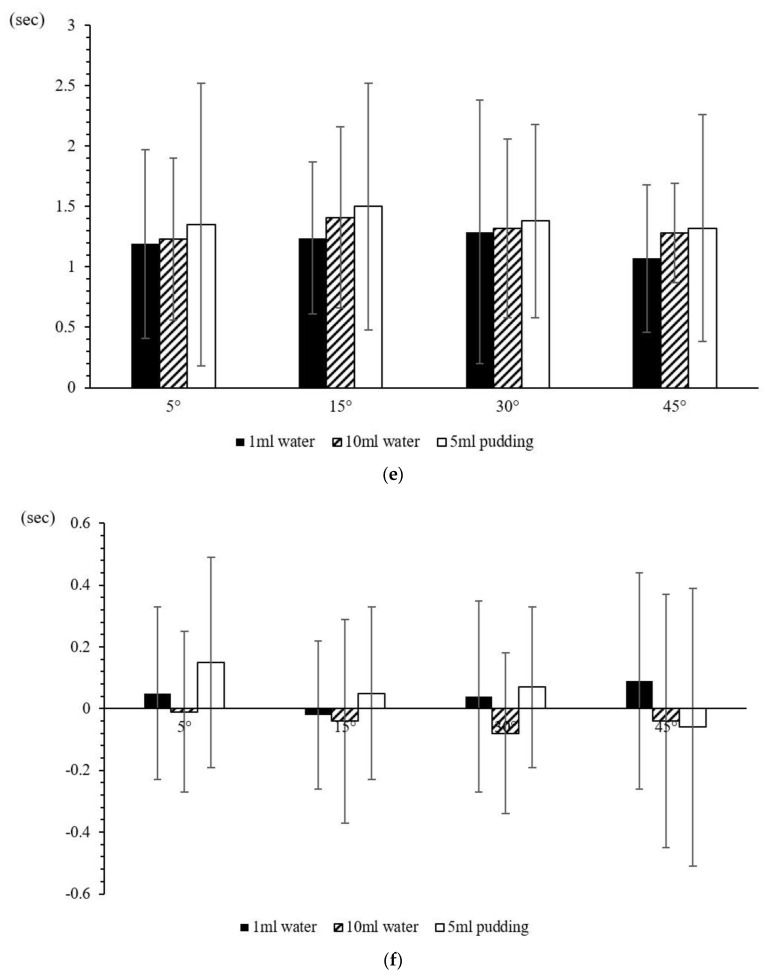

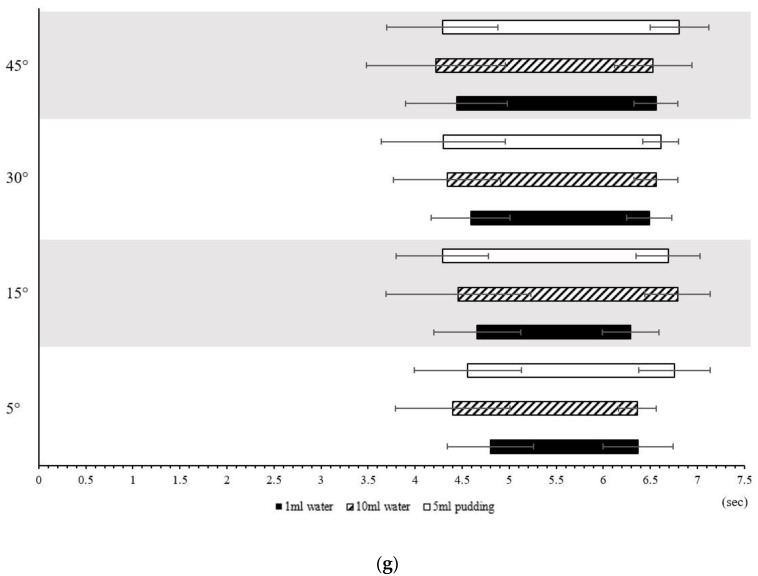
Comparisons among four different trunk inclinations and three different food types: (**a**) D_sEMG_; (**b**) SAD; (**c**) TET; (**d**) FSR amplitude; (**e**) D2DEF; (**f**) onset latency; (**g**) duration between FSR onset–offset. * *p* < 0.05.

Three participants were determined to be incomplete. Therefore, the data of 30 participants (28.6 ± 7.1 years) were further analyzed (Table 3, Figure 4). Significant differences were found among angles only in SAD (*p* = 0.045) and TET (*p* = 0.037). Post hoc exams revealed significance differences between the angles of 5° and 45° (*p* = 0.036 for SAD; *p* = 0.009 for TET).

Significant differences were found among food patterns in D_sEMG_ (*p* < 0.000), SAD (*p* < 0.000), TET (*p* = 0.048), and D2DEF (*p* = 0.043), with most differences being between 1 mL water and 5 mL pudding, and between 10 mL water and 5 mL pudding (Table 4). The negative OL shows that the participants had an earlier onset of the thyroid cartilage movement. No significant differences were found among food patterns in OL (*p* = 0.075) and FSR amplitude (*p* = 0.116). No significant interaction was observed between angle and food patterns whatsoever.

Table 5 shows the frequencies of signals triggered within the combinations of inclination angles and food types. Because the study consisted of healthy participants, the frequency of triggered warnings (yellow or red lights) was relatively low. In the 1 mL water tests, piecemeal swallowing or unsafe respiratory phase did not occur in any combination of conditions because of the minute volume. In the 10 mL water tests with 45° inclination, the warnings triggered were four times yellow and three times red; no warning was triggered with other inclination angles. As in the 5 mL pudding tests, the warnings triggered were eight times yellow and twelve times red with 45° inclination only.

## 4. Discussion

The respiratory–swallow telecare system developed in this study successfully detects various indicators of swallowing efficiency (i.e., submentalis muscle activity, apnea time, thyroid cartilage excursion, presence of piecemeal deglutition, and the breathing patterns before and after swallowing) and instantaneously alerts a caregiver/health care provider to check the positioning and safety of the service recipient. It is especially suitable for use in nursing homes and assisted living facilities. In addition to providing warning signals for on-site and remote personnel, this system can also record the respiratory–swallow coordination information of a service recipient for each meal, compile the relevant data, and upload such data to a remote database. Continuous in-home monitoring can provide extensive and sensitive data that capture the subtle behavioral changes and health information of at-risk individuals [23]. The unsafe conditions during the 45° inclination in this study successfully triggered warnings onsite and remotely. Remote healthcare providers can calculate the ratio of red, yellow, and green lights associated with a service recipient, adjust parameter standards to meet individual needs, or screen for early signs of dysphagia. The collected information can also be used to develop swallowing training programs. For individuals with dysphagia caused by diseases or disorders, the system can be customized to meet individual needs, thus providing safer feeding solutions and improving quality of life.

During feeding, trunk positioning directly affects respiratory–swallow coordination and feeding safety, especially for individuals with disabilities, but this problem is often neglected in practice. Because different types of care recipients have different means of compensating for swallowing (e.g., older adults with sarcopenic dysphagia [24], individuals with stroke or cerebral palsy with spasticity or spasm in the oral or pharyngeal muscles [25,26,27], and individuals with parkinsonism [22]), a caregiver may be unaware of the effects of body positioning on swallowing and breathing. When a care recipient cannot clearly express their feelings or actively change their body position during the feeding process, they are exposed to risks relating to the feeding process. With regard to the recommended angle of inclination during feeding, the results of the present study indicated that a care recipient can swallow more easily when they are sitting upright (5°) than when they are leaning backwards (45°). The recorded thyroid cartilage excursion and apnea time were significantly longer when the 45° inclination was adopted. This finding indicated that the participants required more time to return their thyroid cartilages back to their original positions after swallowing when the 45° inclination was adopted. This phenomenon is usually observed during the early stage of dysphagia [28]. In addition, the feedback from participants indicated that many of them felt discomfort and were concerned about choking when they were in the 45° inclination. The results also revealed significant differences in food content concerning numerous variables. Swallowing pudding required a significantly longer time than swallowing 1 mL of water during submentalis activity, thyroid cartilage excursion, and the second deflection.

However, the literature does not provide consistent recommendations on the appropriate positioning angle for feeding or assisted eating. The present study examined participants who had normal oral motor function and airways (including the laryngeal vestibule) that were closed during swallowing. Other studies with participants with dysphagia or cerebral palsy have controversial findings [12,27,29,30]. These inconsistencies can be related to oral motor dysfunction severity, neuromuscular compensation, body–head position interactions, food texture (liquid, barium solution, or bolus), amount of food consumed, the swallowing method that was used (single or piecemeal), and the evaluation method that was used [12,24,27,28,29,30,31,32]. Furthermore, the hip joint angle during a 45° inclination (e.g., a wheelchair with a tilt-in-space function can maintain the hip at a 90° angle, but the hip joint angle of a reclining wheelchair may be approximately 135°) may affect the comfort level experienced by a participant. A study highlighted that the risk of aspiration can increase unless the optimal position for an individual is selected [33].

The present study still has limitations. First, although the autodetection program with a customized algorithm has been verified previously [9], the criteria for triggering alerts that were applied in the present study have not been verified clinically, and further data collection and clinical comparisons have yet to be performed and validated. Second, the portable device is suitable for institutional and home use, but the throat belt and nasal cannula must be properly worn under instructions for accurate detection. Third, the present study focused on noninvasive measures for achieving swallowing comfort and efficiency, but the residuals after swallowing and the occurrence of aspiration cannot be precisely detected unless a VFSS or fiberoptic endoscopy is performed [34]. Fourth, coughing and talking have many phenotypes that demonstrate considerable variability; therefore, if there was a cough or talk during data collection, the particular trial was aborted. Fifth, because different types of care recipients adopt different strategies to compensate for the swallow, the current autodetection criteria for triggering alerts target common swallowing problems (e.g., piecemeal and non-EXP/EXP breathing pattern). In a practical setting, individualized criteria with greater accuracy can be used to assess swallowing status. It is possible to make customized adjustments for the individual swallowing characteristics of different users in the future.

## 5. Conclusions

The COVID-19 pandemic underlines the importance of telecare. This respiratory–swallow telecare system provides healthcare providers/caregivers a useful and applicable form of remote monitoring and alerting for residents in care facilities. Essential swallowing information of each meal can also be recorded and uploaded to cloud-based platforms for further assessment. With the further development of the relevant algorithms, this system can serve as a pipeline for early screening for swallowing dysfunction. The results can provide references for proper feeding positions for older adults or individuals with disabilities.

## Figures and Tables

**Table 1:** Variables about the respiration and swallowing signals.

Variables	Definition	Calculation
D_sEMG_	Duration of submentalis muscle EMG activity	E1 to E3
SAD	Swallowing apnea duration via nasal airflow	A1 to A2
OL	Onset latency between sEMG and the thyroid cartilage movement	F1 to E1
TET	Total excursion time of the thyroid cartilage excursion	F1 to F3
D_2DEF_	Duration of 2nd deflection in the W-shaped response of the thyroid cartilage	F2 to F3
FSR Amplitude	Thyroid movement	
D_sEMG_ onset/offset time	Onset/offset time of sEMG	E1/E3
FSR onset/offset time	Onset/offset time of FSR	F1/F3
Resp_phase	respiratory phases before/after SAD	State before A1/state after A2 (i.e., EXP/EXP, EXP/INP, INP/EXP, and INP/INP)

**Table 2:** Definition of the risk levels.

Risk Level	Events		Definition
Level 1 (Red alert)	PMD	Piece-meal deglutition	TET > T ^a^ and FSR Amplitude < d ^a^
	L_SAD	Long apnea duration	SAD > T ^a^
Level 2 (Yellow alert)	L_Onset	Long onset latency between sEMG and thyroid cartilage excursion	OL > (1/2)T ^a^
	L_TET	Long total excursion time of thyroid cartilage	TET > T ^a^
	L_ D_2DEF_	Long duration of second deflection in the W-shaped response of the thyroid cartilage	D_2DEF_ > (1/2)T ^a^
	L_ D_sEMG_	Long duration of submental muscle EMG activity	D_sEMG_ > T ^a^
	U_Resp_phase	Unsafe respiratory phases before/after SAD	None-EXP/EXP phases before A1 and after A2

^a^ Note. T: a predefined time threshold, d: a predefined pressure threshold.

**Table 3:** Descriptive statistics of each variable under different inclination angles and food types.

Variables	D_sEMG_ (s)	SAD (s)	TET (s)	D_2DEF_ (s)	FSR Amplitude (V)	OL (s)
Conditions	Mean (SD)					
5° vs. 1 mL water	1.66 (0.82)	0.58 (0.18)	2.12 (0.79)	1.19 (0.78)	0.51 (0.15)	0.05 (0.28)
5° vs. 10 mL water	1.72 (0.64)	0.64 (0.20)	2.13 (0.59)	1.23 (0.67)	0.47 (0.15)	−0.01 (0.26)
5° vs. 5 mL pudding	2.17 (1.17)	0.62 (0.21)	2.43 (1.36)	1.35 (1.17)	0.52 (0.31)	0.15 (0.34)
15° vs. 1 mL water	1.60 (0.49)	0.59 (0.29)	2.02 (0.61)	1.24 (0.63)	0.49 (0.14)	−0.02 (0.24)
15° vs. 10 mL water	1.58 (0.59)	0.63 (0.15)	2.22 (0.62)	1.41 (0.75)	0.49 (0.15)	−0.04 (0.33)
15° vs. 5 mL pudding	2.06 (0.84)	0.65 (0.17)	2.46 (1.10)	1.50 (1.02)	0.49 (0.13)	0.05 (0.28)
30° vs. 1 mL water	1.59 (0.63)	0.57 (0.13)	2.27 (0.92)	1.29 (1.09)	0.49 (0.18)	0.04 (0.31)
30° vs. 10 mL water	1.49 (0.54)	0.64 (0.14)	2.27 (0.70)	1.32 (0.74)	0.50 (0.16)	−0.08 (0.26)
30° vs. 5 mL pudding	1.88 (0.73)	0.72 (0.13)	2.42 (0.82)	1.38 (0.80)	0.54 (0.16)	0.07 (0.26)
45° vs. 1 mL water	1.46 (0.43)	0.60 (0.12)	2.24 (0.68)	1.07 (0.61)	0.48 (0.21)	0.09 (0.35)
45° vs. 10 mL water	1.51 (0.60)	0.69 (0.09)	2.34 (0.74)	1.25 (0.41)	0.52 (0.16)	−0.04 (0.41)
45° vs. 5 mL pudding	1.86 (0.81)	0.77 (0.21)	2.54 (1.06)	1.32 (0.94)	0.55 (0.15)	−0.06 (0.45)

**Table 4:** Differences of variables between different inclination angles and food types.

	Inclination			Condition			Interaction
	*p*	Bonferroni		*p*	Bonferroni		*p*
			*p*			*p*	
D_sEMG_	0.516	5° vs. 15°	0.368	0.000 *	1 mL water × 10 mL water	0.786	0.459
		5° vs. 30°	0.904		1 mL water × 5 mL pudding	0.000 *	
		5° vs. 45°	0.485		10 mL water × 5 mL pudding	0.000 *	
		15° vs. 30°	0.367				
		15° vs. 45°	0.161				
		30° vs. 45°	0.402				
SAD	0.045 *	5° vs. 15°	0.651	0.000 *	1 mL water × 10 mL water	0.069	0.115
		5° vs. 30°	0.383		1 mL water × 5 mL pudding	0.000 *	
		5° vs. 45°	0.036 *		10 mL water × 5 mL pudding	0.016 *	
		15° vs. 30°	0.684				
		15° vs. 45°	0.109				
		30° vs. 45°	0.228				
TET	0.037 *	5° vs. 15°	0.402	0.048 *	1 mL water × 10 mL water	0.292	0.129
		5° × 30°	0.096		1 mL water × 5 mL pudding	0.047 *	
		5° vs. 45°	0.009 *		10 mL water × 5 mL pudding	0.212	
		15° vs. 30°	0.127				
		15° vs. 45°	0.044 *				
		30° vs. 45°	0.517				
FSR Amplitude	0.628	5° vs. 15°	0.568	0.116	1 mL water vs. 10 mL water	0.787	0.441
		5° vs. 30°	0.742		1 mL water vs. 5 mL pudding	0.125	
		5° vs. 45°	0.669		10 mL water vs. 5 mL pudding	0.062	
		15° vs. 30°	0.276				
		15° vs. 45°	0.188				
		30° vs. 45°	0.784				
D_2DEF_	0.166	5° vs. 15°	0.064	0.043 *	1 mL water × 10 mL water	0.052	0.823
		5° vs. 30°	0.083		1 mL water × 5 mL pudding	0.037 *	
		5° vs. 45°	0.093		10 mL water × 5 mL pudding	0.362	
		15° vs. 30°	0.063				
		15° vs. 45°	0.083				
		30° vs. 45°	0.080				
OL	0.328	5° vs. 15°	0.095	0.075	1 mL water × 10 mL water	0.062	0.057
		5° vs. 30°	0.087		1 mL water × 5 mL pudding	0.803	
		5° vs. 45°	0.254		10 mL water × 5 mL pudding	0.056	
		15° vs. 30°	0.623				
		15° vs. 45°	0.995				
		30° vs. *45°	0.793				

* *p* < 0.05. α Alpha value for Bonferroni was 0.05.

**Table 5:** Signals triggered within different combinations of inclination angles and food types. (Number of participants = 30; repetitions for each combination = 3).

Inclination	5°	15°	30°	45°	5°	15°	30°	45°	5°	15°	30°	45°
**Food types**	**1 mL water**				**10 mL water**				**5 mL pudding**			
Total trials in each combination	90	90	90	90	90	90	90	90	90	90	90	90
Frequency of lights triggered (%)												
**Base level:** Green indicators	90 (100%)	90 (100%)	90 (100%)	90 (100%)	90 (100%)	90 (100%)	90 (100%)	83 (92.2%)	90 (100%)	90 (100%)	90 (100%)	70 (77.8%)
**Level 2:** Yellow indicators	0 (0%)	0 (0%)	0 (0%)	0 (0%)	0 (0%)	0 (0%)	0 (0%)	4 (4.4%)	0 (0%)	0 (0%)	0 (0%)	8 (8.9%)
**Level 1:** Red indicators	0 (0%)	0 (0%)	0 (0%)	0 (0%)	0 (0%)	0 (0%)	0 (0%)	3 (3.3%)	0 (0%)	0 (0%)	0 (0%)	12 (13.3%)

## Data Availability

Not applicable.

## Abbreviations

VFSS	Videofluoroscopic swallow study
FSR	Force sensing resistor
D_sEMG_	Duration of surface electromyography (for submentalis activity)
SAD	Swallowing apnea duration
OL	Onset latency
TET	Total excursion time
D_2DEF_	Duration of 2nd deflection
